# Novel 3D human trophoblast culture to explore *T. cruzi* infection in the placenta

**DOI:** 10.3389/fcimb.2024.1433424

**Published:** 2024-08-06

**Authors:** Sofia Apodaca, Marco Di Salvatore, Arturo Muñoz-Calderón, María de los Ángeles Curto, Silvia A. Longhi, Alejandro G. Schijman

**Affiliations:** Laboratorio de Biología Molecular de la Enfermedad de Chagas, Instituto de Investigaciones en Ingeniería Genética y Biología Molecular "Dr. Héctor Torres" (INGEBI), Consejo Nacional de Investigaciones Científicas y Técnicas (CONICET), Buenos Aires, Argentina

**Keywords:** congenital Chagas disease, placental tropism, human trophoblast, three-dimensional microtissue model, *Trypanosoma cruzi* infection, quantitative PCR, syncytiotrophoblast

## Abstract

**Introduction:**

Human trophoblastic cell lines, such as BeWo, are commonly used in 2D models to study placental *Trypanosoma cruzi* infections. However, these models do not accurately represent natural infections. Three-dimensional (3D) microtissue cultures offer a more physiologically relevant in vitro model, mimicking tissue microarchitecture and providing an environment closer to natural infections. These 3D cultures exhibit functions such as cell proliferation, differentiation, morphogenesis, and gene expression that resemble in vivo conditions.

**Methods:**

We developed a 3D culture model using the human trophoblastic cell line BeWo and nonadherent agarose molds from the MicroTissues® 3D Petri Dish® system. Both small (12–256) and large (12–81) models were tested with varying initial cell numbers. We measured the diameter of the 3D cultures and evaluated cell viability using Trypan Blue dye. Trophoblast functionality was assessed by measuring β-hCG production via ELISA. Cell fusion was evaluated using confocal microscopy, with Phalloidin or ZO-1 marking cell edges and DAPI staining nuclei. *T. cruzi* infection was assessed by microscopy and quantitative PCR, targeting the EF1-α gene for *T. cruzi* and GAPDH for BeWo cells, using three parasite strains: VD (isolated from a congenital Chagas disease infant and classified as Tc VI), and K98 and Pan4 (unrelated to congenital infection and classified as Tc I).

**Results:**

Seeding 1000 BeWo cells per microwell in the large model resulted in comparable cellular viability to 2D cultures, with a theoretical diameter of 408.68 ± 12.65 μm observed at 5 days. Functionality, assessed through β-hCG production, exceeded levels in 2D cultures at both 3 and 5 days. *T. cruzi* infection was confirmed by qPCR and microscopy, showing parasite presence inside the cells for all three tested strains. The distribution and progression of the infection varied with each strain.

**Discussion:**

This innovative 3D model offers a simple yet effective approach for generating viable and functional cultures susceptible to *T. cruzi* infection, presenting significant potential for studying the placental microenvironment.

## 1 Introduction

*Trypanosoma cruzi*, the causative agent of Chagas disease (CD), one of the most neglected tropical diseases (NTDs) in the world, affects around 7 million people in 21 endemic countries of America as well as in non-endemic countries due to increased migration (Chagas disease in Latin America: an epidemiological update based on 2010 estimates, 2015; Antinori et al., 2017; Organización Panamericana de la Salud, 2020). It is estimated that globally more than two million women of reproductive age are affected by *T. cruzi* infection, and 1–10% of fetuses carried by infected mothers are born with CD (Oliveira et al., 2010; Carlier and Truyens, 2015; Carlier et al., 2015).

Transplacental transmission of *T. cruzi* causes congenital CD (cCD) in 4–8% of chronic CD women`s offspring, and even if cCD does not occur, pregnancy outcome may exhibit increased risk of preterm birth, low-birth weight and stillbirth (Torrico et al., 2004; Liempi et al., 2016).

The 63rd World Health Assembly urged governments to establish algorithms for early diagnosis of NTDs in newborns, with emphasis in cCD (WHA63.20 Chagas disease: control and elimination, 2010). Although cCD infants can develop severe clinical forms, most are asymptomatic, so unlikely to be diagnosed and treated unless the infection is specifically tested for (Messenger and Bern, 2018; Picado et al., 2018). If untreated, cCD infants are at risk of disabling and life-threatening chronic pathologies later in life (Picado et al., 2018).

To infect the developing fetus, *T. cruzi* trypomastigotes (TCT), must cross the trophoblasts, which form the first fetal tissue of the placental barrier. This epithelium forms a covering of mononuclear villous cytotrophoblasts (CT) lying beneath syncytiotrophoblasts (ST), which are in direct contact with maternal blood. After gestational week 20, CT cells diminish and the nuclei of ST cells group together forming nodes. This restructuring favors metabolic exchange due to the formation of thin cytoplasmatic areas devoid of nuclei, so that fetal capillaries come close to the ST and the placental membrane transforms into a thinner barrier. At this stage, parasite invasion may be facilitated causing cCD.

It has been proposed that maternal bloodstream TCTs in the intervillous space infects ST, CT and fetal connective tissue of the villous stroma invading the different cell types. Trypomastigotes differentiate into intracellular amastigotes (ICA), which proliferate and after a certain number of replications differentiate again into TCTs, which can invade the fetal capillaries reaching the fetus (Duaso et al., 2010; Carlier et al., 2012; Liempi et al., 2016).

It was reported that some mothers transmit Chagas disease through successive pregnancies and even across generations of congenitally infected women. This phenomenon is known as family clustering of vertical transmission (Burgos et al., 2007; Bisio et al., 2011; Kemmerling et al., 2019). The mechanisms underlying cCD family clustering remain to be elucidated; the interplay between parasite genetic diversity and placental factors appear to be involved (Kemmerling et al., 2019). Bloodstream TCT burden in pregnant women, which varies with the parasite strains, is a key element determining transmission (Burgos et al., 2007; Bisio et al., 2011; Bua et al., 2012; Bua et al., 2013). *T. cruzi* populations are classified in seven discrete typing units (DTUs, TcI-TcVI and Tcbat), on the basis of immunological, biochemical and genetic markers with intra-DTU diversity (Zingales et al., 2012; Brenière et al., 2016). Different clones exhibit differential tissue tropism and virulence (Macedo and Pena, 1998; Macedo et al., 2004) and certain haplotypes have been associated with cCD (Carlier and Truyens, 2015; Herrera et al., 2019). Furthermore, tissue tropism to the placenta of different *T*. *cruzi* strains has been described, particularly in the murine model (Andrade, 1982); Colombiana strain (Tc I) presents a high incidence of placental parasitism (98%) compared to Y strain (Tc II) that only infects 17% of the placentas (Andrade, 1982).

Human trophoblastic cell lines, such as BeWo, have been frequently used as 2D host models for placental *T. cruzi* infection studies (Liempi et al., 2015; Droguett et al., 2017). Despite it has been demonstrated that *T. cruzi* induces trophoblast differentiation, denoted by expression of β-hCG and syncyntin as well as by cell fusion forming STs (Liempi et al., 2015; Droguett et al., 2017), 2D cultures are poor representatives of natural trophoblast infections.

Three-dimensional microtissue cultures (3DMT, spheroids) have been developed as novel *in-vitro* models, physiologically relevant, as they can mimic the microarchitecture of tissues providing an environment like that found in natural infections (Shamir and Ewald, 2014). Biological functions of 3D cultures that approach those of *in vivo* situations include cell proliferation, differentiation, morphogenesis, and gene expression (Smalley et al., 2006; Yamada and Cukierman, 2007; Shamir and Ewald, 2014). A 3D placental MT model has been developed to recreate the human placental environment and to perform nanotoxicity tests (Muoth et al., 2016). When cultured in 3D, trophoblast cells express markers associated with STs’ differentiation and produce placental hormones (Silberstein et al., 2021).

Previous studies using the murine experimental model have shown that a *T. cruzi* clone isolated from a cCD case exhibited a different degree of infectivity to placental tissues compared to another clone that is not transmissible through the placenta (Juiz et al., 2017).

This study aims to construct a 3D microtissue model with BeWo cells and explore infectivity differences among *T. cruzi* strains, specifically those associated or not with vertical transmission.

## 2 Materials and methods

### 2.1 Trophoblast cell culture

The human trophoblast cell line BeWo was used. Cultures were maintained with DMEM-F12 medium (Gibco, Grand Island, NY) supplemented with 5% inactivated SFB (Internegocios S.A., Mercedes, Buenos Aires, Argentina), 2mM L-glutamine (Gibco, Grand Island, NY), 100 IU/ml penicillin/100 μg/ml streptomycin (Sigma-Aldrich, St. Louis, MO) in a humid atmosphere containing 5% CO_2_ at 37.5°C. Cultures were held to approximately 80% confluence and treated with 0.25% Trypsin-0.04% EDTA (Gibco, Grand Island, NY) to disrupt and subculture.

### 2.2 Trypanosoma cruzi cultures

The strains VD, K98 and Pan4 of *T. cruzi* were used. VD (Tc VI) is a clone obtained from an argentinean cCD patient (Risso et al., 2004) and K98 (Tc I) (Solana et al., 2002) is a clone derived from the non-lethal myotropic CA-I strain that is not transmissible through placenta, isolated from an argentinean chronic cardiac CD patient (Solana et al., 2002). For the experiments carried out with K98 strain, a transfected clone expressing the fluorescent green protein (K98-GFP) was used (Miranda et al., 2015).

Pan4 is a clone derived from a strain originally isolated from a 32-year-old patient from the Arraijan district (Panamá) in 2006, kindly donated by Dr Antonio Osuna de Albornoz and used a high virulent strain in tissues others than placenta (De Pablos et al., 2011).

These parasite stocks were cultured at their epimastigote stage in LIT medium supplemented with 10% SFB, 20mg/mL hemin and 100 IU/ml penicillin/100 μg/ml streptomycin (Sigma-Aldrich, St. Louis, MO) at 28°C until stationary phase to increase the percentage of parasites in the metacyclic trypomastigote stage. At this point, the parasites were placed in contact with BeWo cell cultures to obtain trypomastigotes in the culture medium after 5–7 days, with which the infection tests were carried out.

### 2.3 Three-dimensional cultures

For three-dimensional (3D) cultures the MicroTissues® 3D Petri Dish® system (Sigma‐Aldrich®, St. Louis, MO) were used. This system consists of an autoclavable silicone mold that is used to generate agarose 3D Petri Dish® with non-stick microwells where three-dimensional cultures will be formed. Two models were used: 12–256 small spheroids (cat. Z764000–6EA) and 12–81 Large spheroids (cat. Z764019–6EA) and proceeded according to the manufacturer’s recommendations. Briefly, 2% agarose in 0.9% NaCl solution was autoclaved and allowed to cool to 60–70°C. Each autoclaved silicone mold was filled with 500 µl of molten agarose and after allowing it to solidify, the silicone molds were flexed with forceps to facilitate the agarose 3D Petri Dish separate from the mold. These agarose molds were placed in a 12-wells plate and two incubations were carried out in 10% SFB cell culture medium for 15 minutes to equilibrate them.

Each cell suspension tested was placed in the micromolds, in a final volume of 190 µL and after 15 minutes, 2.5mL of cell culture medium were added.

Suspensions of BeWo cells at various concentrations were examined to identify the optimal conditions for forming uniform spheroids that were not constrained by well size and did not exceed 500 µm in diameter. This size limitation is based on findings by Hirschhaeuser et al (Hirschhaeuser et al., 2010), which indicate that central secondary necrosis typically occurs in tumor spheroid cultures exceeding 500 µm in size.,

Starting with the concentrations estimated by the manufacturers, 3375 cells per microwell for both models, 1000 for Large molds and 316 for small molds were tested.

The 3D cultures were observed using a ZEISS “AXIO Vert.A1” inverted microscope equipped with a ZEISS “Axiocam 202 mono” camera (Carl Zeiss AG, Oberkochen, Germany). The perimeter of the spheroids was assessed using Fiji (Schindelin et al., 2012), and the theoretical diameter was reported as if they were spherical.

Using the LIVE/DEAD Cell Imaging Kit (Thermo Scientific, Rockford, IL, USA), spheroids of 1000 cells per microwell of the Large model were analyzed after 3 days of seeding after incubation with the dye for 3 hours. Using the Zeiss LSM 880 Confocal Microscope (Carl Zeiss AG, Oberkochen, Germany), a z-stack was performed taking images every 1.6 um in the z axis. The excitation wavelengths used were 488 nm and 543 nm, and the detection ranges were 494.32–558.26 nm and 632.51–735 nm, respectively. A 20X objective with a numerical aperture of 0.8 (Plan-Apochromat 20x/0.8 M27) and a magnification of 1.2X were used.

The images were processed with ZEN (blue edition, version 3.5.093.00004) software (Carl Zeiss AG, Oberkochen, Germany) to perform 3D reconstructions and with Fiji for maximum intensity projection.

### 2.4 Live and dead cells in the different models

The proportion of live and dead cells in the cultures was measured using the Trypan Blue dye. For this, after 3 or 5 days of culture, the spheroids were removed from their molds up and down with a 1000 µL pipette, transferred to 1.5 mL tubes and washed 3 times with PBS, allowing them to decant by gravity between each wash. After washing, the spheroids were incubated with Trypsin-EDTA for 30 min at 37°C, shaking the tubes every 10 min.

Subsequently, 1 mL of culture medium was added, and pipetting continued until no clumps were discernible. The mixture was then centrifuged for 8 minutes at 500 rpm and subsequently resuspended in a final volume of 150 µL.

In 2D cultures, trypsin treatment lasted for 8 minutes, while the following steps of the procedure remained unchanged. The resulting cell suspension was mixed in a 1:1 ratio with Trypan Blue dye and examined under the microscope using a Neubauer chamber. Three independent cultures were conducted for each model for 3 and 5 days (2D, 3D small, and 3D Large), ensuring that in all instances, over 100 total cells were counted.

### 2.5 β-hCG measurement

To assess culture functionality, the concentration of the Beta subunit of Chorionic Gonadotropin (β-hCG) in the culture supernatant was measured both in the presence and absence of forskolin stimulation. Forskolin induces trophoblast cell fusion and has been demonstrated to trigger this process in the BeWo cell line (Orendi et al., 2010). Measuring β-hCG under these conditions serves as an indicator of functionality in cultures, allowing for comparison to the forskolin stimulus. Equivalent cell quantities were utilized in both 2D and 3D cultures of varying sizes. After one day, the culture medium was refreshed, and forskolin was added to half of the cultures at a final concentration of 20 µM. Supernatant samples were collected on days 3 and 5, with the culture medium renewed each time, and promptly frozen at -80°C until quantification. In all cases the volume of culture medium was 2.5 mL.

The quantification of β-hCG was conducted through ELISA, employing a Beckman Coulter Access Total ß-hCG kit (Beckman Coulter Inc., CA, USA), following the manufacturer’s recommendations.

### 2.6 Immunofluorescence microscopy

Cultures with or without forskolin were removed from the molds at day 5, washed with PBS and incubated with 4% paraformaldehyde for 3h. After this time, three 15-min washes were performed with PBS and 1 mL of 10% w/v sucrose in PBS was added and incubated overnight. Using Disposable Base Molds (Fisher Scientific, Hampton, NH, EEUU), the spheroids were embedded in 10% gelatin plus 10% sucrose in PBS. Gelatin plugs were adhered to cork squares using Tissue Plus O.C.T. Compound (Fisher Scientific, Hampton, NH, EEUU). Isopentane was used to freeze them, adding dry ice to achieve a temperature of -50°C, and the blocks were submerged for one minute and then stored at -80°C until cutting. Sections were made in a cryostat, 20um thick, and placed on Superfrost Plus Microscope Slides (Fisher Scientific, Hampton, NH, EEUU).

Prior to immunolabeling, samples were washed with PBS, permeabilized with 0.2% Triton X-100 for 5 min, and then submerged in PBS for at least 5 min. Solutions of Phalloidin Alexa Fluor™ 546 1X, ZO-1 ALEXA FLUOR 647 (AB_2663167) 1 µg/mL antibody (Thermo Scientific, Rockford, IL, USA), anti-*T*. *cruzi* mouse serum 1/1000 (Benatar et al., 2015) in PBS- 0.5% BSA were used, and incubated for 90 minutes at room temperature, protected from light. Alexa Fluor 488 conjugated goat anti-mouse IgG (AB_2536161, Thermo Scientific, Rockford, IL, USA) was used as secondary Ab 1/200 in PBS-0,1% BSA and incubated for 60 minutes at room temperature. Finally, the slides were washed once with PBS 0.05% Tween 20 and twice with PBS during 5 min each.

The specimens were mounted using VECTASHIELD Antifade Mounting Medium with Dapi from Vector Laboratories (Newark, CA, USA). Visualization was performed using a confocal Leica TCS SPE microscope at a 40X magnification with an immersion objective, utilizing 405, 488, 532, and 635 lasers. Three distinct replicates were generated, and photographs of three randomly selected sections were captured.

The fusion radius was determined by calculating 100 times the number of nuclei within syncytia (defined as three or more nuclei lacking zo-1 or actin barrier) to the total number of nuclei.

### 2.7 Infection with different strains of *T. cruzi*

Spheroids of 1000 cells were grown per microwell using the Large mold for 3 days, the culture medium was removed and fresh medium containing trypomastigotes of strains VD, K98 and PAN4 was added at MOI 20:1 and incubated for 48 hours.

Conventional 2D cultures were also put in contact with the same *T. cruzi* strains and MOI described above for 3 hours, washed with PBS 7–10 times and cultured for 48 hours.

To assess infectivity, three replicates were analyzed for each strain using microscopy to visualize the infection. Each replicate consisted of either 81 spheroids or a coverslip for 2D cultures. After fixation, labeling for actin and *T. cruzi* was carried out, excluding the K98-GFP strain.

### 2.8 Quantification of parasite loads in spheroids

Three wells of 2D infected cultures, as previously described, underwent trypsin treatment and were subsequently collected in Eppendorf tubes, pooling the samples for each strain of *T. cruzi.*

Three wells containing infected spheroids, following the described procedure, were transferred to a Falcon tube, creating a pool for each strain. Subsequently, 10 washes were performed to remove parasites that were not located within the spheroids.

DNA was extracted using the High Pure PCR Template Preparation kit (Roche Diagnostics GmbH, Mannheim, Germany) according to the manufacturer’s instructions for tissues.

Comparative parasite loads in 2D and 3D cultures were determined for each strain by means of qPCR using primers for the *T. cruzi* Elongation factor 1α gene (Billaut-Mulot et al., 1996; Alves et al., 2015) (EF-1α, EF-1αfw 5’-GGAGGCATTGACAAGCGGACGAT-3’, EF-1αrv 5´-GATCGTGAACACAGACTTGGGCG-3) and GAPDH for the quantification of human trophoblastic cells (GAPDH fw 5′-GGTCTCCTCTGACTTCAACA-3′ and GAPDH rev 5′-GTGAGGGTCTCTCTCTTCCT-3′).

Quantification was performed utilizing standard curves specific to each set of primers.

For EF-1α gene, the pGEM®-T Easy Vector System (Promega, Madison, Wisconsin, USA) was employed to construct a recombinant plasmid containing a single EF-1α gene copy, and serial 1:10 dilutions were prepared, ranging from 8x10^7 to 8x10^2 copies/µl to build a standard curve for *T.cruzi* quantification.

For GAPDH, BeWo cells were enumerated in a Neubauer chamber, and DNA extraction was carried out. Subsequently, 5 serial dilutions at 1:10 were prepared, beginning at 6.66 x 10^5 cells/mL to construct the standard curve for quantifying BeWo cells.

The qPCR was performed in the QG 9600 PeetLab Real Time Thermocycler (China) using the FG POWER SYBR GREEN PCR mix (Thermo Scientific, Rockford, IL, USA), 0.3 µM of primers and 1/100 dilution of the extracted DNA. Cycling included 94°C for 5 minutes, 40 cycles of 94°C for 30 seconds + 58°C for 30 seconds, and finally, a Melting curve from 75 to 95°C.

Using the standard curve corresponding to each gene, the parasite copy number per BeWo cell and the number of BeWo cells in each infected spheroid sample were calculated.

### 2.9 Statistics

Statistical comparisons were conducted using one-way ANOVA followed by the Tukey test for multiple-group comparisons, three replicates per experiment were performed and are presented as the median with standard deviation.

For the analysis of the parasitic load per BeWo cell, Student’s T-test was used to compare the 2D and 3D models. A p-value < 0.05 was considered statistically significant.

## 3 Results

### 3.1 3D culture growth profile

**Figure 1:**
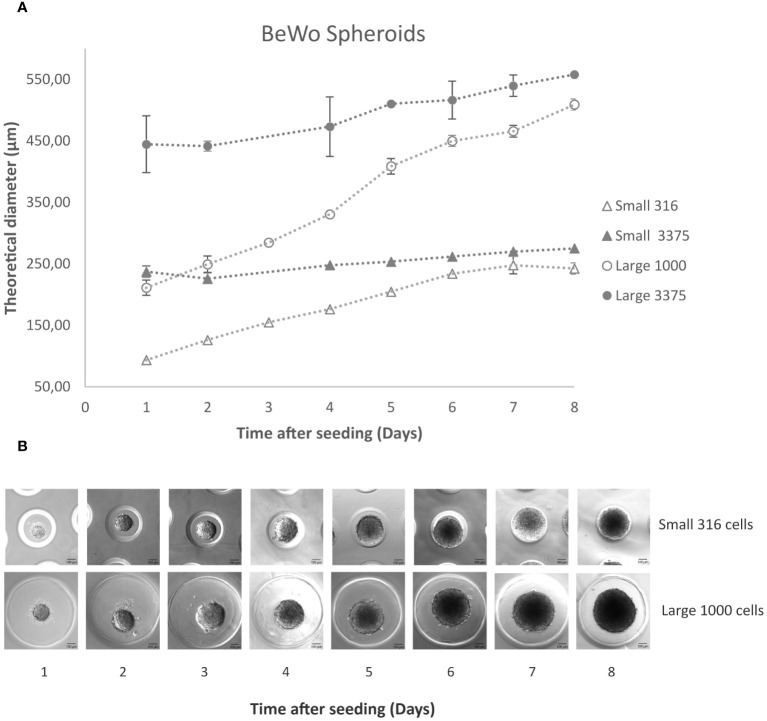
BeWo spheroids. **(A)** Using the 3D Petri Dish system, 3375 and 316 cells per microwell in the small model (full and empty triangles respectively) and 3375 and 1000 cells per microwell in the Large model (full and empty circles respectively) were seeded according to the manufacturer’s protocol. The graph shows the average of 3 measurements and their standard deviation for each condition. **(B)** Sequential images using the inverted microscope illustrating the daily progression of results after seeding 316 and 1000 cells per microwell in the Small and Large mold respectively. Scale bar: 100 μm.

Small and Large molds were used for seeding. In **Figure 1A**, the growth profile of both Small and Large spheroids, consisting of 3375 cells per microwell, is depicted over an 8-day period. Spheroids in both molds were observed to form as early as day 1 post-seeding. By day 4, the Large spheroids surpassed the critical diameter of 500 µm, whereas the Small ones did not exceed the critical diameter due to limitations imposed by the microwell size.

Subsequently, a reduced number of cells—1000 cells per Large microwell and 316 cells per small microwell—were tested. It was noted that the spheroids formed uniformly, and their growth was not restricted by the microwell diameter, yet they did not surpass 500 µm even after 5 days (**Figures 1A, B**).

Consequently, it was decided to proceed with these spheroids for further experimentation.

### 3.2 Viability and functionality

**Figure 2:**
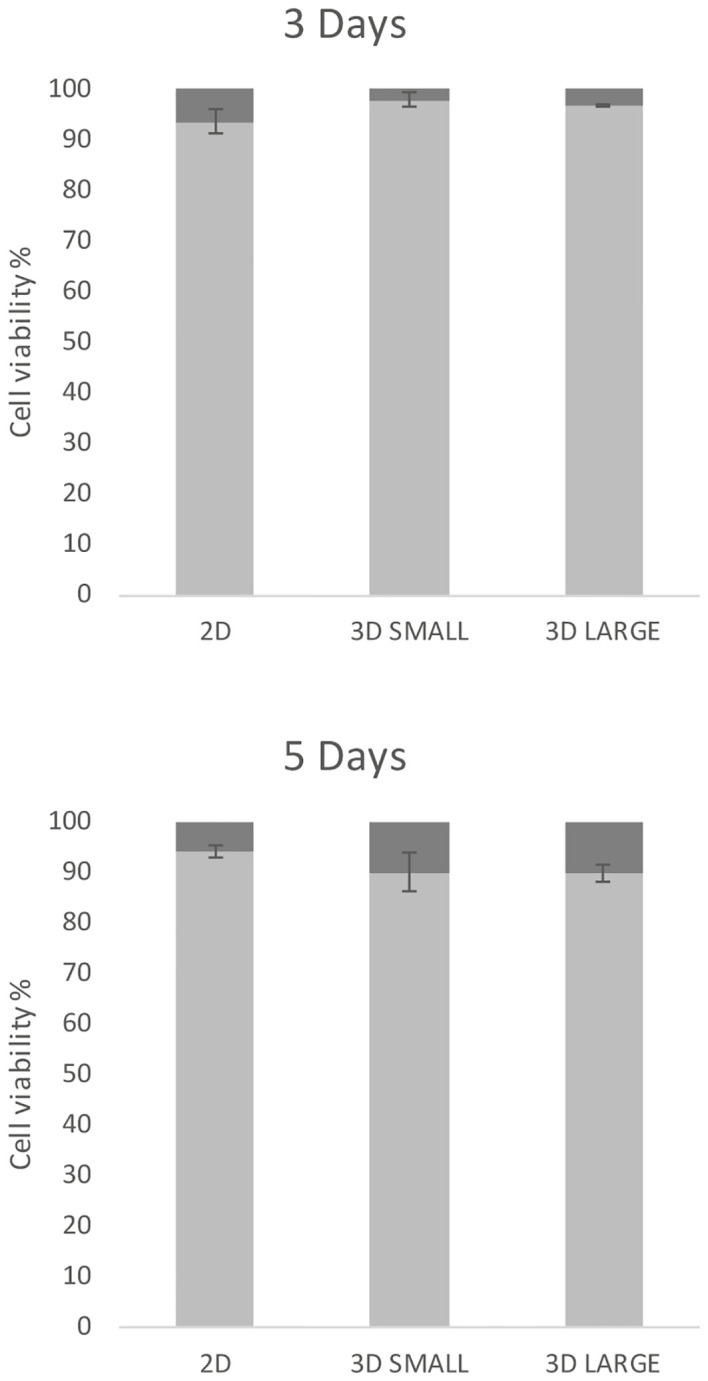
Cell viability. 2D and 3D cultures, initiated with the same number of cells, were grown for 3 days and 5 days, then dissociated and stained with Trypan blue. The bars represent the percentage of live (light gray) and dead (dark gray) cells along with the standard deviation.

In order to set up *T.cruzi* infection experiments, the cellular viability of spheroids was firstly evaluated in comparison to 2D cultures by disassembling cell cultures at 3 and 5 days. In **Figure 2**, the percentages of viable and non-viable cells are illustrated for 2D and 3D Small and Large models. The initial seeding involved 81,000 cells per well (equivalent to 316 and 1000 cells in the Small and Large models, respectively), with or without a micromold.

Across all three culture types, a substantial percentage of live cells was observed at both 3 days (exceeding 93%) and 5 days, with a marginal decrease in viability (approximately 90% of live cells). No significant differences were noted for each model at both incubation time points, suggesting comparability among these models.

**Figure 3:**
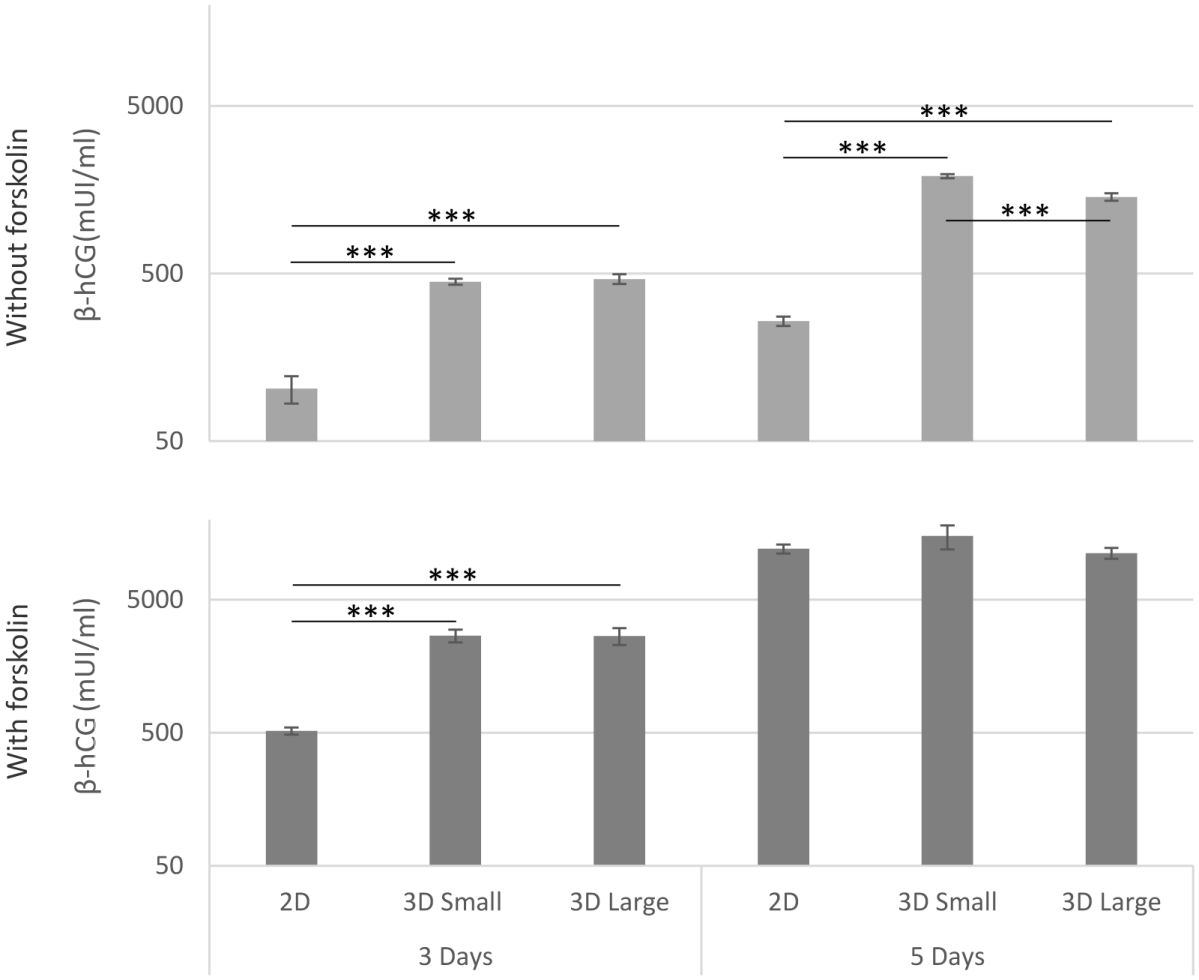
β-hCG production. The release of β-hCG into the culture medium in the different models was quantified using ELISA, both with and without forskolin stimulation at days 3 and 5 after seeding. (***p<0.001).

The assessment of β-hCG levels in the culture medium served as an indicator of trophoblast functionality across different models, both with and without forskolin stimulation (**Figure 3**). In the absence of stimuli, noticeably elevated levels of β-hCG were evident in both spheroid sizes (Small and Large) and at both time points (3 and 5 days), surpassing the levels observed in 2D cultures, confirming a functional trophoblast microtissue. For cultures treated with forskolin, significant differences were noted at 3 days. However, by the fifth day, all cultures exhibited elevated levels of β-hCG in the supernatant (*** p<0.001).

**Figure 4:**
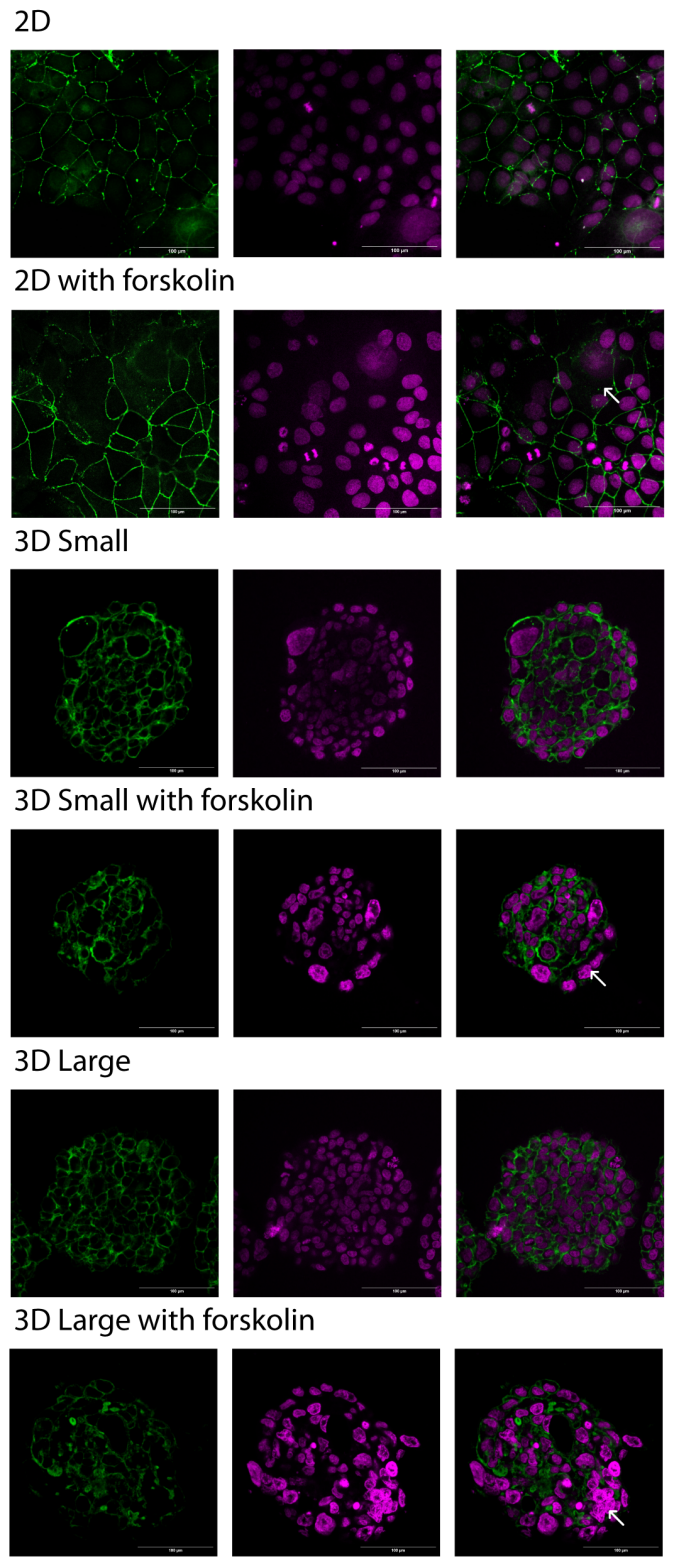
Trophoblast fusion. 2D and 3D cultures, with and without forskolin, were fixed at 5 days. Cryostat sections were prepared, and immunofluorescent labeling with Phalloidin or Zo-1 (in green) for membrane visualization and DAPI (in magenta) for nuclei was performed. Confocal Microscopy images were taken. The images on the right column show the merge of both channels. The white arrow points to a syncytium. Scale bar: 100 µm.

The ST displays a multinucleated structure resulting from the fusion of cytotrophoblasts. To measure the extent of fusion, cultures were incubated for five days before being prepared for fluorescence microscopy. Actin was labeled using Phalloidin, while ZO-1 was utilized to mark cell junctions. Subsequently, the radius of cell fusion was calculated for quantitative assessment (**Figure 4** and **Supplementary Figure 1**). In the absence of forskolin, the fusion ratios were below 8% for all models. However, in its presence, the fusion ratios ranged from 25% to 50%, with no significant differences observed between the models (**Supplementary Figure 1**). Based on these findings, the Large model was selected for *T. cruzi* infection experiments. **Supplementary Figure 2** shows the 3D reconstruction of the Large spheroid model used in subsequent infection assays. Large spheroids were easier to manipulate when preparing samples for microscopy, and their viability remained unaffected at both 3 and 5 days.

### 3.3 Infection with *T. cruzi* strains

*T. cruzi* infection experiments were carried out in absence of forskolin, given that functionality measured by β-hCG production exhibited a substantial increase compared to 2D cultures.

**Figure 5:**
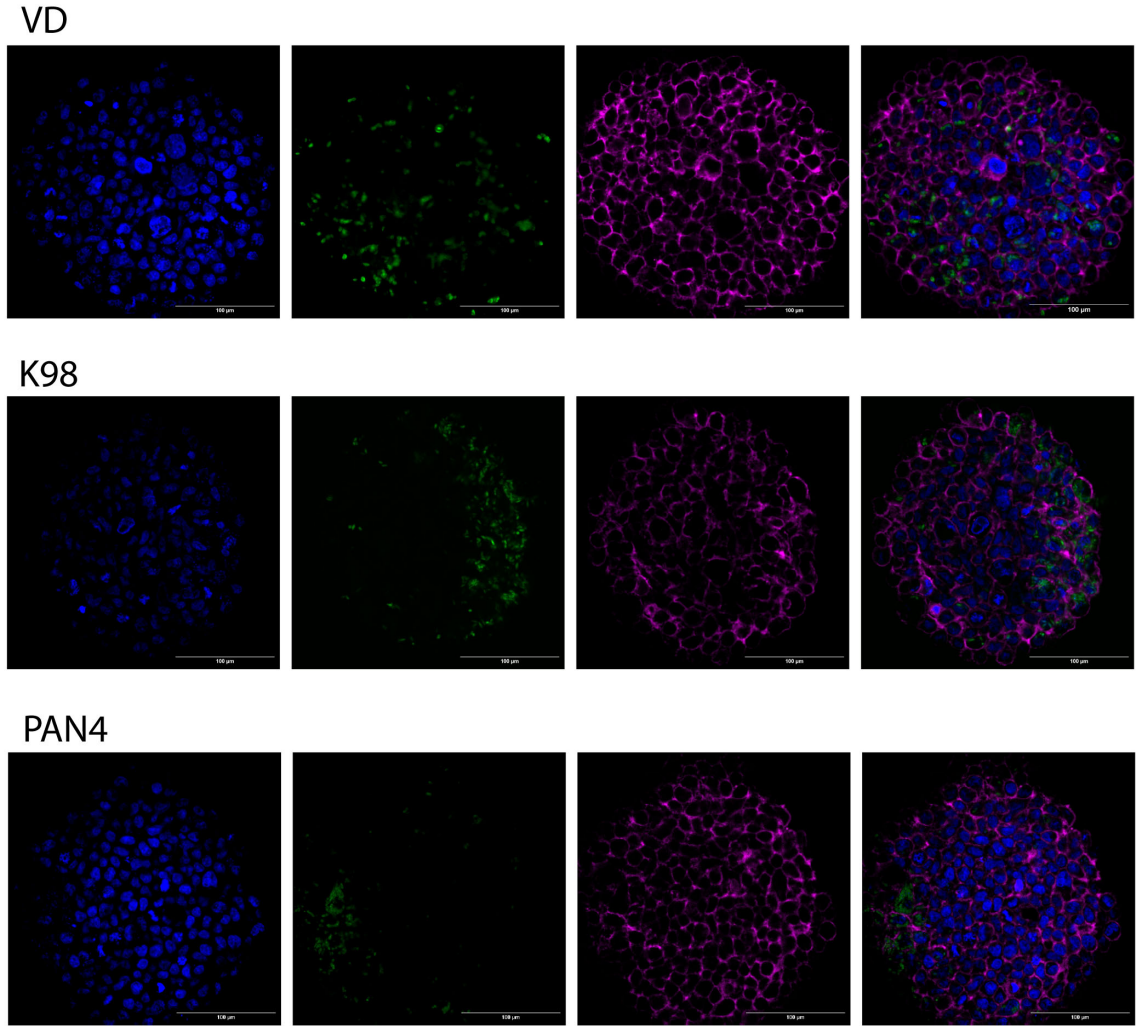
Infection of 3D cultures by congenital and non-congenital *T. cruzi* strains. Three-day-old spheroids were exposed to *T. cruzi* trypomastigotes from K98, VD and PAN4 stocks. From left to right, the images display examples of infected spheroids, showing nuclei in blue, parasites in green, phalloidin labeling in magenta, and the merge of channels. Scale bar: 100 µm.

Three-day-old 3D Large cultures were exposed to *T. cruzi* trypomastigotes for 48 hours. Following this exposure, the cultures were fixed and labeled using immunofluorescence. Using all three parasitic stocks (VD, K98-PtrexGFP, and PAN4), the infection proved successful, and intracellular amastigotes were observable. This was demonstrated either by the expression of green fluorescent proteins or by labeling with an anti-*T. cruzi* antibody, as shown in **Figure 5** (one example per strain) and in **Supplementary Figures 3–5** for VD, K98-PtrexGFP, and PAN4, respectively.

Parasite load quantification was conducted through qPCR using primers specific for a *T. cruzi* conservative gene and human cells. The outcomes are presented as the number of *T. cruzi* target gene DNA copies per BeWo cell. For VD strain, a significant difference was observed between 2D and 3D models: in 2D cultures, the EF-1α per cell ratio was 0.09 ± 0.05, while in 3DMT it was 2.30 ± 0.69 (p < 0.01). For K98 strain, the difference between 2D and 3DMT was smaller, with the EF-1α per BeWo cell ratio being 0.12 ± 0.03 for 2D cultures and 0.28 ± 0.07 (p < 0.05) for 3DMT cultures.

**Figure 6:**
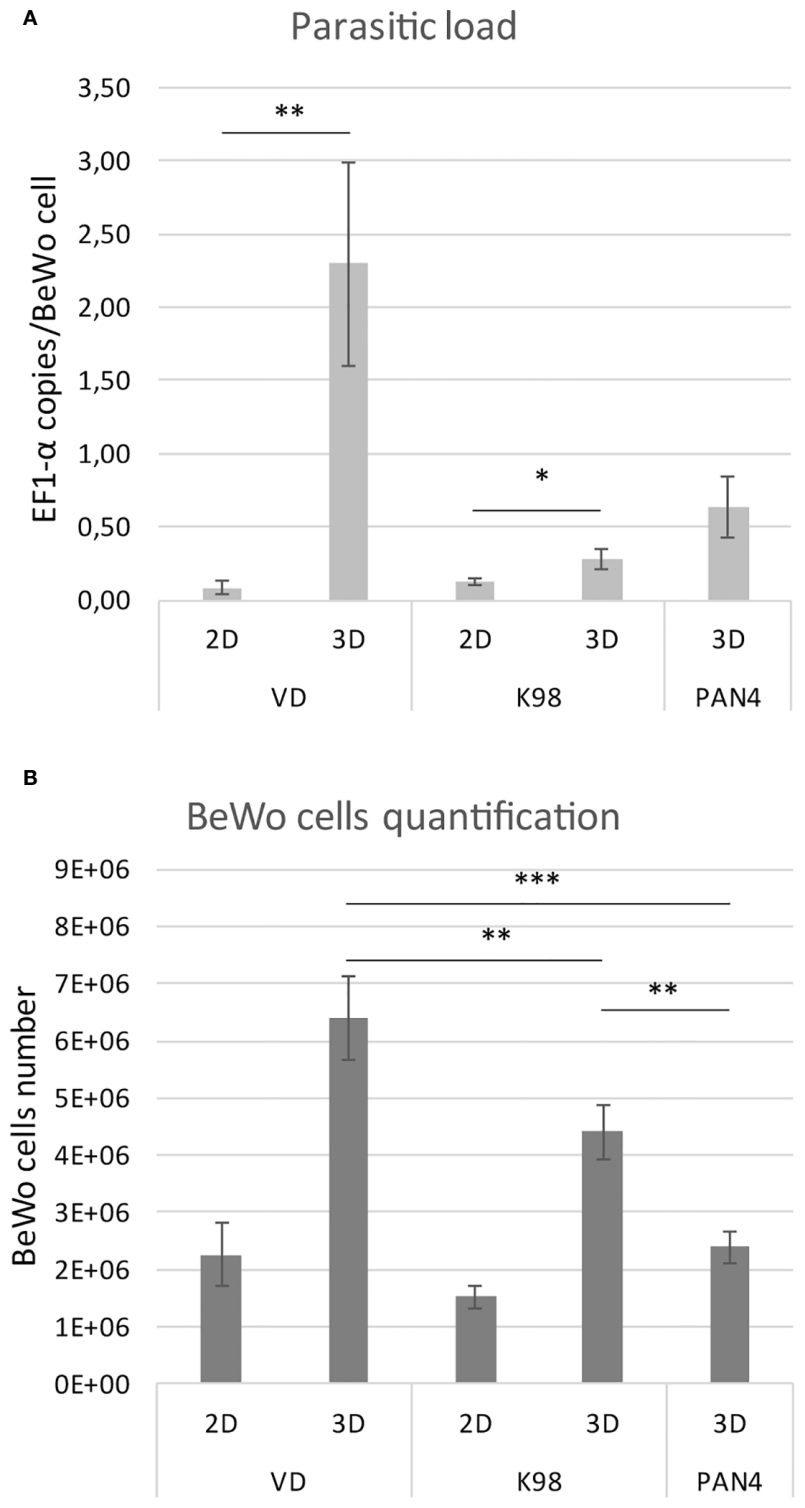
**(A)**. Parasitic loads of *T.cruzi* strains in BeWo spheroids. Quantification by qPCR of the parasite load expressed as copies of *T. cruzi* EF1-α per BeWo cell. **(B)**. BeWo cells quantification. Comparison of numbers of infected BeWo cells by means of a qPCR test targeting the human GAPDH gene. A standard curve made of serial dilutions of DNA extracted from counted BeWo cells was used for PCR quantification. The results are presented as the mean with the SD. *p<0,05; **p< 0,01; ***p<0,001.

In the case of PAN4, infection in 3DMT resulted in a ratio of 0.63 ± 0.21 copies of EF-1α per BeWo cell (**Figure 6A**). Furthermore, we compared the number of BeWo cells in infection experiments using different *T. cruzi* strains by means of a qPCR test targeting the human GAPDH gene. The 3D culture infected with the congenital VD strain showed the highest cell count, followed by K98, and finally PAN4 (**Figure 6B**).

## 4 Discussion

Spheroids, which are dense cell aggregates flourishing in a non-adherent 3D environment, mimic their native microenvironment by fostering intercellular interactions and secreting extracellular matrix components (Fennema et al., 2013; Zanoni et al., 2016).

In our study, we observed that with an initial seeding of 1000 cells per Large microwell and 316 per Small microwell, the theoretical diameter did not surpass 500 µm after 5 days. This aligns with the estimated limit value to prevent the formation of a necrotic center (Hirschhaeuser et al., 2010). In fact, cell viability in 3D BeWo cultures remained relatively stable compared to 2D cultures. Furthermore, in the absence of forskolin induction, spheroids expressed β-hCG, indicating trophoblast functionality. This observation held true for both small and Large spheroids at 3 and 5 days, with elevated β-hCG levels compared to those observed in 2D BeWo cultures. This result is consistent with a study comparing 3DMT of trophoblasts, including the BeWo cell line, where an increase in β-hCG production was observed in the 3D cultures compared to the 2D ones (Stojanovska et al., 2022).

In principle, either of these two culture models could have been used for the infection assays, but Large models were chosen as they were easier to manipulate when assembling samples for microscopy, being easily observable to the naked eye.

Although β-hCG levels measured in 3D cultures were higher than in 2D cultures even in the absence of forskolin stimulation, trophoblast fusion was scarcely observed in 3D with respect to 2D cultures, being in all cases without stimulus, less than 8%. In the presence of forskolin, fusion radio ranged between 25 and 50%, with no significant differences between the models. Therefore, unless forskolin is utilized, this model might be limited in accurately representing the third trimester of pregnancy. In contrast, Silberstein’s study demonstrated the spontaneous fusion of trophoblastic cells from the JEG-3 line when co-cultured with human brain microvascular endothelial cells attached to microcarrier beads in a rotating biorreactor (Silberstein et al., 2021).

Since functionality, measured by β-hCG production, exhibited a substantial increase without the requirement for supplemental forskolin in the culture medium, in this work we chose to minimize the artificial manipulation and performed infection experiments without this stimulus.

Despite their potential, the use of spheroids in exploring infectious diseases and host-parasite interactions has been limited.

Our study focuses on establishing 3D human trophoblast microtissues susceptible to various *T. cruzi* strains. In contrast to a previous investigation involving Jeg-3 cells, which exhibited reduced susceptibility to *T. cruzi* in 3D cultures (Silberstein et al., 2021), the use of BeWo trophoblasts revealed that different *T. cruzi* strains were able to infect the spheroids. Intracellular amastigotes were observed by microscopy for the three strains tested (**Figure 5** and **Supplementary Figures 3–5**) In the case of the VD strain, a wider distribution of the infection was observed, with parasites detected even in the core of the spheroids. For K98, the distribution appeared to be more limited to areas near the spheroid surface. For PAN4, a greater parasite burden was observed in the infected cells, suggesting a higher rate of amastigote replication. Additionally, the distribution of PAN4 showed high green fluorescence, forming patches on the surface of the spheroids and dispersed parasites reaching the core.

We performed qPCR to estimate the parasite load per BeWo cell, which resulted in significant differences between 2D and 3DMT cultures for each strain tested. Moreover, for strain VD, this difference was more pronounced in 3DMT, which is in agreement with previous *in-vivo* findings in the murine model, showing that VD exhibited higher placental tropism than K98 (Juiz et al., 2017), suggesting that these trophoblastic spheroids could better represent *in vivo* conditions of infectivity than 2D models. Indeed, when culture in 2D, the K98 strain produced a higher percentage of infected cells than VD (unpublished data). Regarding the PAN4 strain, if we consider that it may have the same dosage of EF-1α copies than K98, since both strains belong to the same DTU, we can infer that in 3DMT cultures, the number of parasites per BeWo cell is higher than for K98. This is consistent with the fact that PAN4 is considered a highly virulent strain (De Pablos et al., 2011).

Moreover, when we estimated the number of BeWo cells in the infected cultures using the human GAPDH gene as a surrogate marker in qPCR experiments, we found that VD-infected spheroids harbored the highest number of host cells. This suggests that the congenital VD strain might induce the proliferation of trophoblast cells. Further studies will be necessary to demonstrate the influence of *T. cruzi* infection on the cell cycle of BeWo cells in 3D cultures. This effect could be related to the epithelial turnover previously reported in infected 2D BeWo cultures and human placental chorionic villi explants (Liempi et al., 2016).

The congenital transmission mechanism of *T. cruzi* may include not only invading trophoblasts but migrating between them. Rodriguez and colleagues expanded their analysis to *T. cruzi* strains from vertically infected children in myocyte spheroids, revealing a highly migratory phenotype (Rodríguez et al., 2020). Conversely, an isolate from an infected mother, who did not transmit the infection to her children, exhibited significantly less migration. The examination of migration patterns in BeWo spheroids has the potential to provide valuable insights.

It has been shown that 3D trophoblast cultures exhibit a differential transcription pattern compared to 2D cultures, with significant up-regulations in canonical pathways and biological processes such as immune response, angiogenesis, response to stimulus, and wound healing (Wong et al., 2019). Therefore, it could be inferred that the interaction with the parasite would be modified, consistently with our results. Transcriptomic studies on the parasite-host interaction in this new model are relevant to further investigate the placental response to the presence/infection of *T. cruzi*.

Our research positions spheroids as helpful tools for studying placental-pathogen interactions.

## Data availability statement

The raw data supporting the conclusions of this article will be made available by the authors, without undue reservation.

## Ethics statement

Ethical approval was not required for the studies on humans in accordance with the local legislation and institutional requirements because only commercially available established cell lines were used.

## Author contributions

SA: Data curation, Formal analysis, Investigation, Methodology, Software, Visualization, Writing – original draft, Writing – review & editing. MDS: Methodology, Visualization, Writing – review & editing. AM-C: Formal analysis, Investigation, Methodology, Visualization, Writing – review & editing. MAC: Methodology, Writing – review & editing. SL: Data curation, Supervision, Validation, Visualization, Writing – original draft, Writing – review & editing. AS: Conceptualization, Formal analysis, Funding acquisition, Investigation, Project administration, Resources, Supervision, Validation, Writing – original draft, Writing – review & editing.
