# Supplementary figures and images for: Novel 3D human trophoblast culture to explore T. cruzi infection in the placenta
Source: Front Cell Infect Microbiol. 2024 Aug 6;14:1433424. doi: 10.3389/fcimb.2024.1433424 (PMC11333438; doi:10.3389/fcimb.2024.1433424)

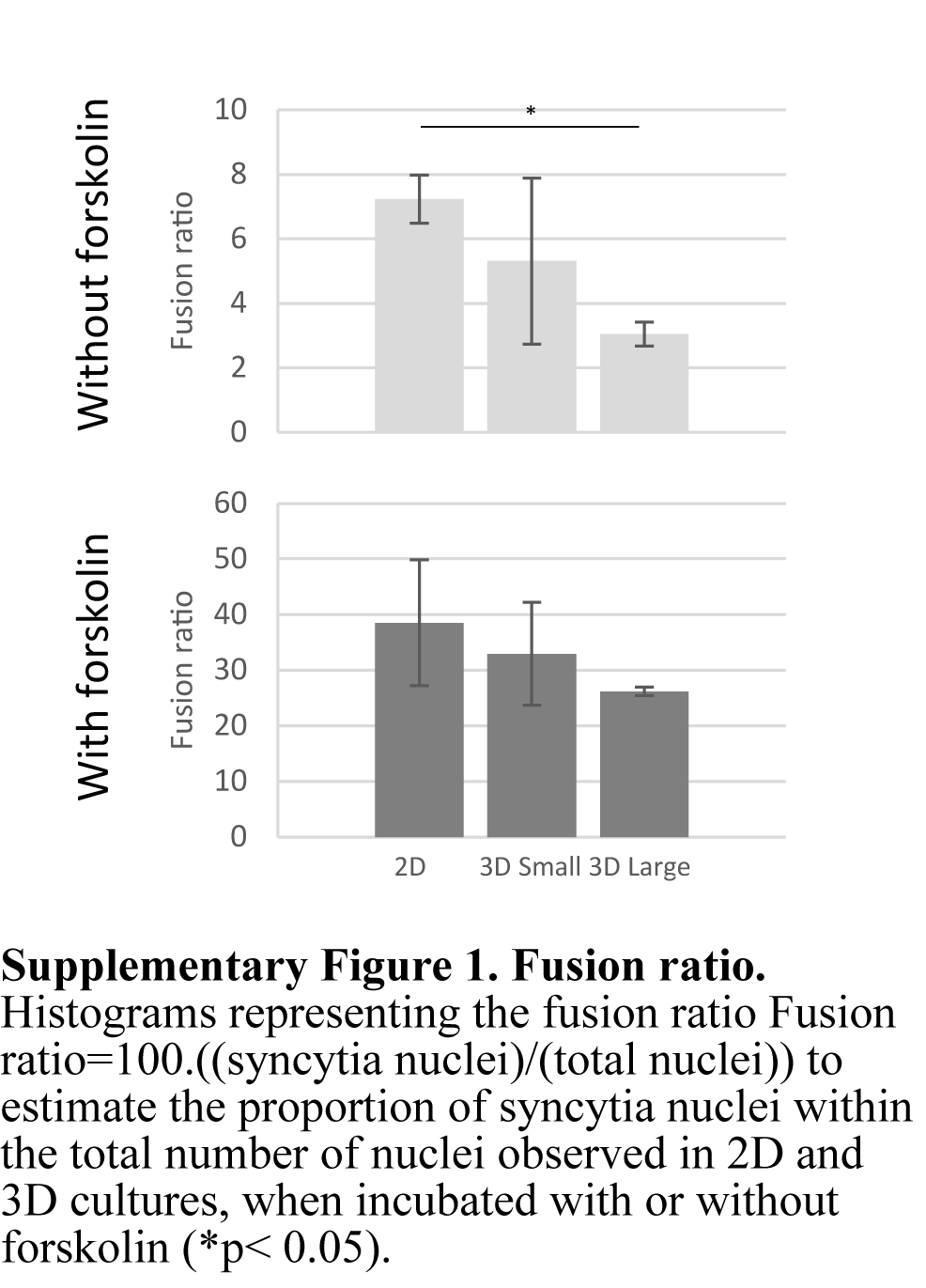

Supplement: Supplementary file 1 [file Image_1.tif]

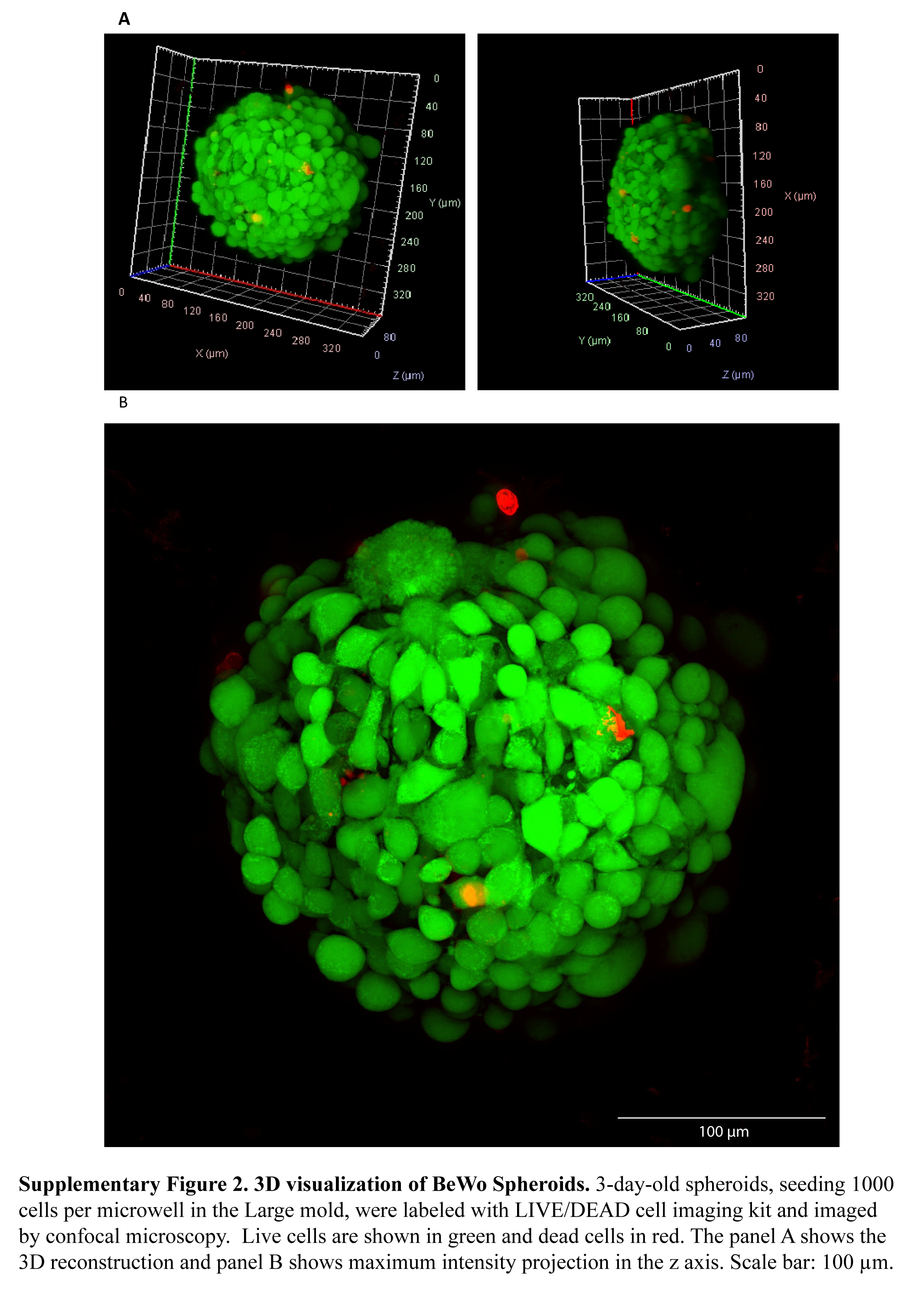

Supplement: Supplementary file 2 [file Image_2.tif]

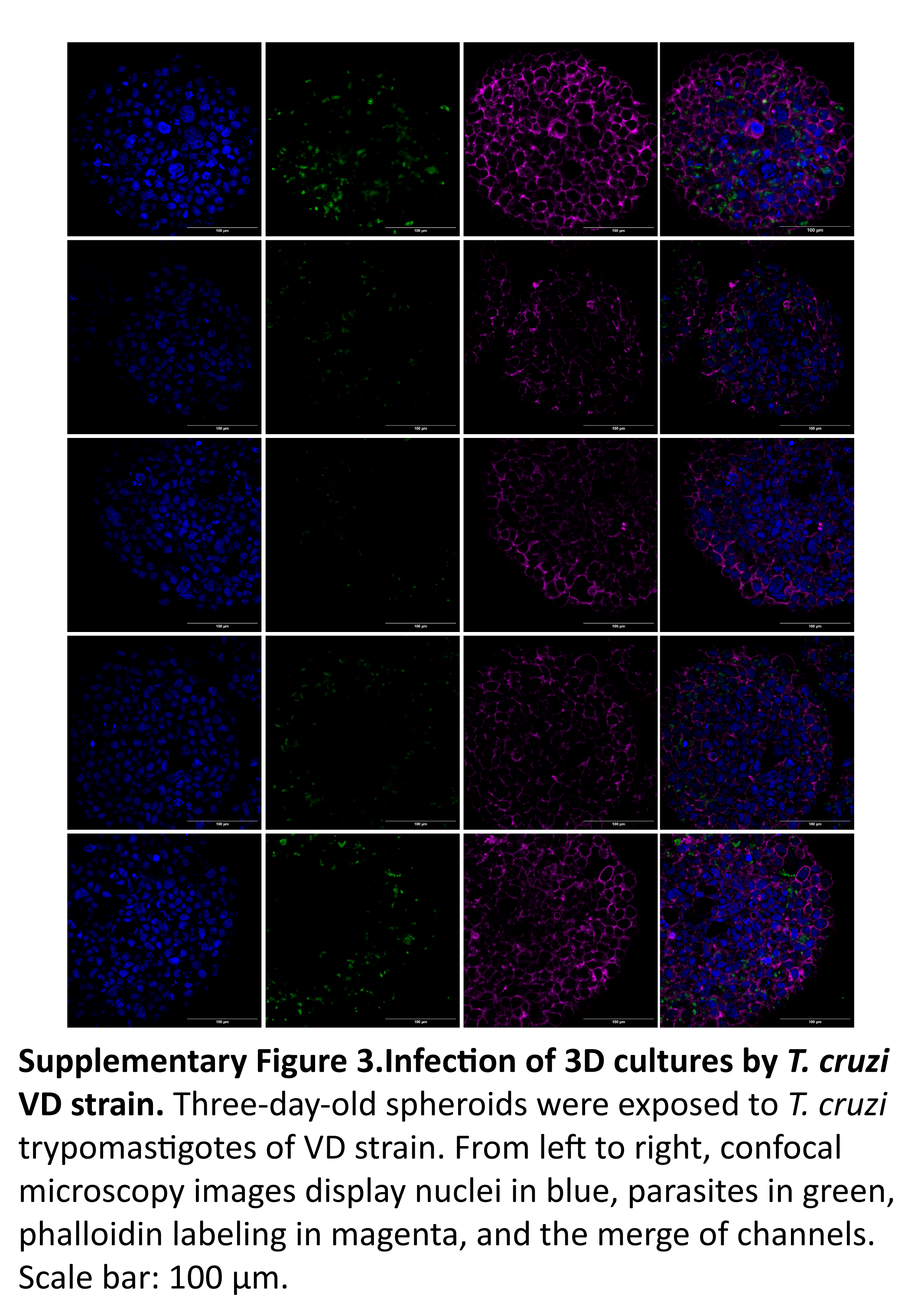

Supplement: Supplementary file 3 [file Image_3.tif]

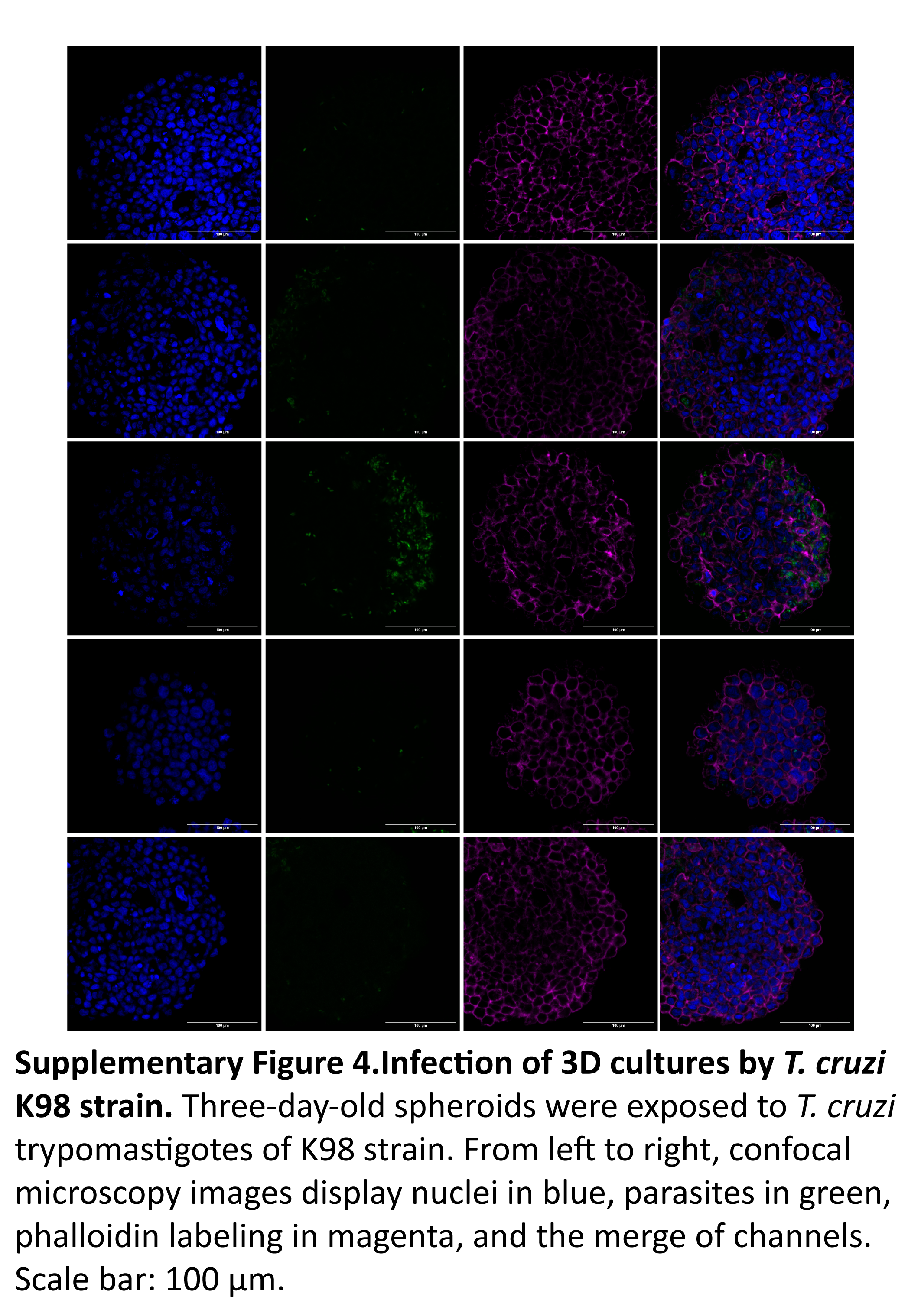

Supplement: Supplementary file 4 [file Image_4.tif]

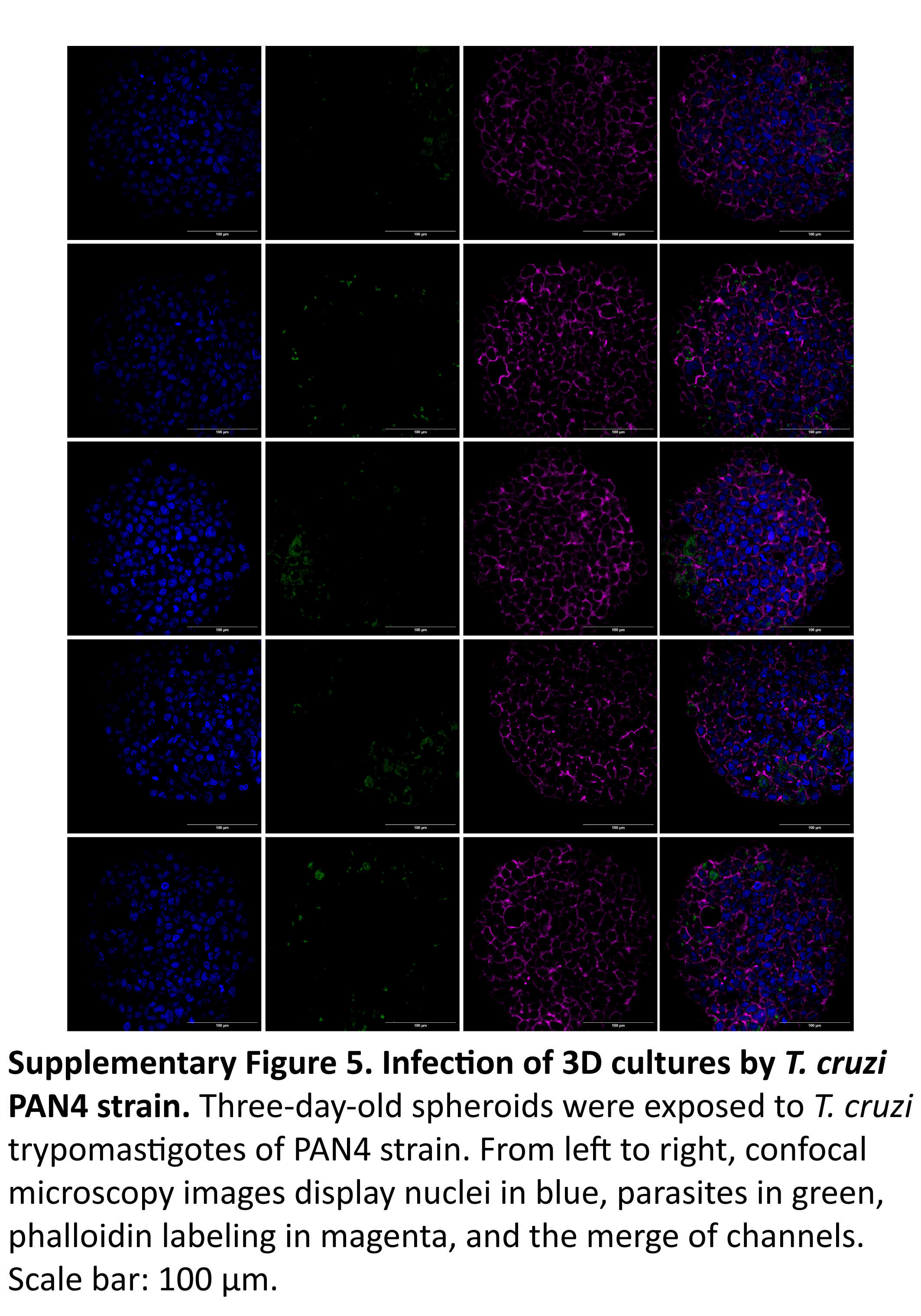

Supplement: Supplementary file 5 [file Image_5.tif]
